# Heart Slices to Model Cardiac Physiology

**DOI:** 10.3389/fphar.2021.617922

**Published:** 2021-02-04

**Authors:** Moustafa H. Meki, Jessica M. Miller, Tamer M. A. Mohamed

**Affiliations:** ^1^ From the Institute of Molecular Cardiology, Department of Medicine, University of Louisville, Louisville, KY, United States; ^2^ Department of Bioengineering, University of Louisville, Louisville, KY, United States; ^3^ Department of Pharmacology and Toxicology, University of Louisville, Louisville, KY, United States; ^4^ Institute of Cardiovascular Sciences, University of Manchester, Manchester, United Kingdom

**Keywords:** heart, cardiotoxicity, cardiomyocytes, iPS-cell derived cardiomyocytes, heart slices

## Abstract

Translational research in the cardiovascular field is hampered by the unavailability of cardiac models that can recapitulate organ-level physiology of the myocardium. Outside the body, cardiac tissue undergoes rapid dedifferentiation and maladaptation in culture. There is an ever-growing demand for preclinical platforms that allow for accurate, standardized, long-term, and rapid drug testing. Heart slices is an emerging technology that solves many of the problems with conventional myocardial culture systems. Heart slices are thin (<400 µm) slices of heart tissue from the adult ventricle. Several recent studies using heart slices have shown their ability to maintain the adult phenotype for prolonged periods in a multi cell-type environment. Here, we review the current status of cardiac culture systems and highlight the unique advantages offered by heart slices in the light of recent efforts in developing physiologically relevant heart slice culture systems.

## Introduction

Before clinical studies, there is a need for reliable experimental systems that can accurately replicate the human heart's physiological environment. Such systems should model altered mechanical loading, heart rate, and electrophysiological properties. The most prevalently used cardiac physiology screening platform are animal models that have limited reliability in mirroring the effects of drugs in human hearts (Liu et al., 2006; Salama and Bett, 2014). Additionally, the use of animal models to create a pharmacokinetic profile of drugs is relatively expensive at the early development stage since large amounts of the drugs are used (Guth et al., 2019). Ultimately, the ideal experimental cardiac model is one that demonstrates high sensitivity and specificity toward various therapeutic and pharmacological interventions while accurately replicating the physiology and pathophysiology of the human heart (Wang et al., 2008).

## *In-Vitro* Modeling of the Human Myocardium

*In-vitro* systems using a controlled culture environment have the unique advantage of quantitatively characterizing drug-related changes at the cellular level with relatively lower cost. These systems’ success depends on how much clinically relevant information can be detected through the system. This includes contractility measurements, protein expression (e.g., gap junction proteins and contractile proteins), accurate transcriptional profile, calcium homeostasis, and electrophysiology. Additionally, the applicability of the system to human cells is of paramount importance. Guth et al. (Guth et al., 2019) summarized the most relevant features and considerations for *in-vivo* drug testing platforms.

Nonetheless, 28% of drug withdrawals are owed to unanticipated cardiotoxicity (Gwathmey et al., 2009). This only shows that current cardiac models are unsuitable for preclinical drug testing and development. To this end, there have been many efforts to develop reliable preclinical models to mimic the human heart tissue. This review will focus on the most relevant cardiac physiology models: isolated adult cardiomyocytes, human induced pluripotent stem cell-derived cardiomyocytes, arterially perfused left ventricular wedge preparations, and subsequently, compare them to heart slices.

Thin heart slices are a relatively new technology that has emerged in recent years and has shown promise in providing a system of adequate complexity and viability to allow for drug screening (Miller et al., 2020). They retain the adult phenotype, including the adult heart’s multicellularity and extracellular structure, and have been produced from human cardiac tissue and large mammals.

While each of these models has its advantages, each model has shortcomings in modeling the physiology/pathophysiology of an adult human heart. This is owed to the heart tissue’s complex environment where multiple cell types interact under chronic neurohormonal stimulation to maintain normal heart homeostasis. It is thus understandable that there are many challenges in reproducing this multiplexed system in culture.

**FIGURE 1:**
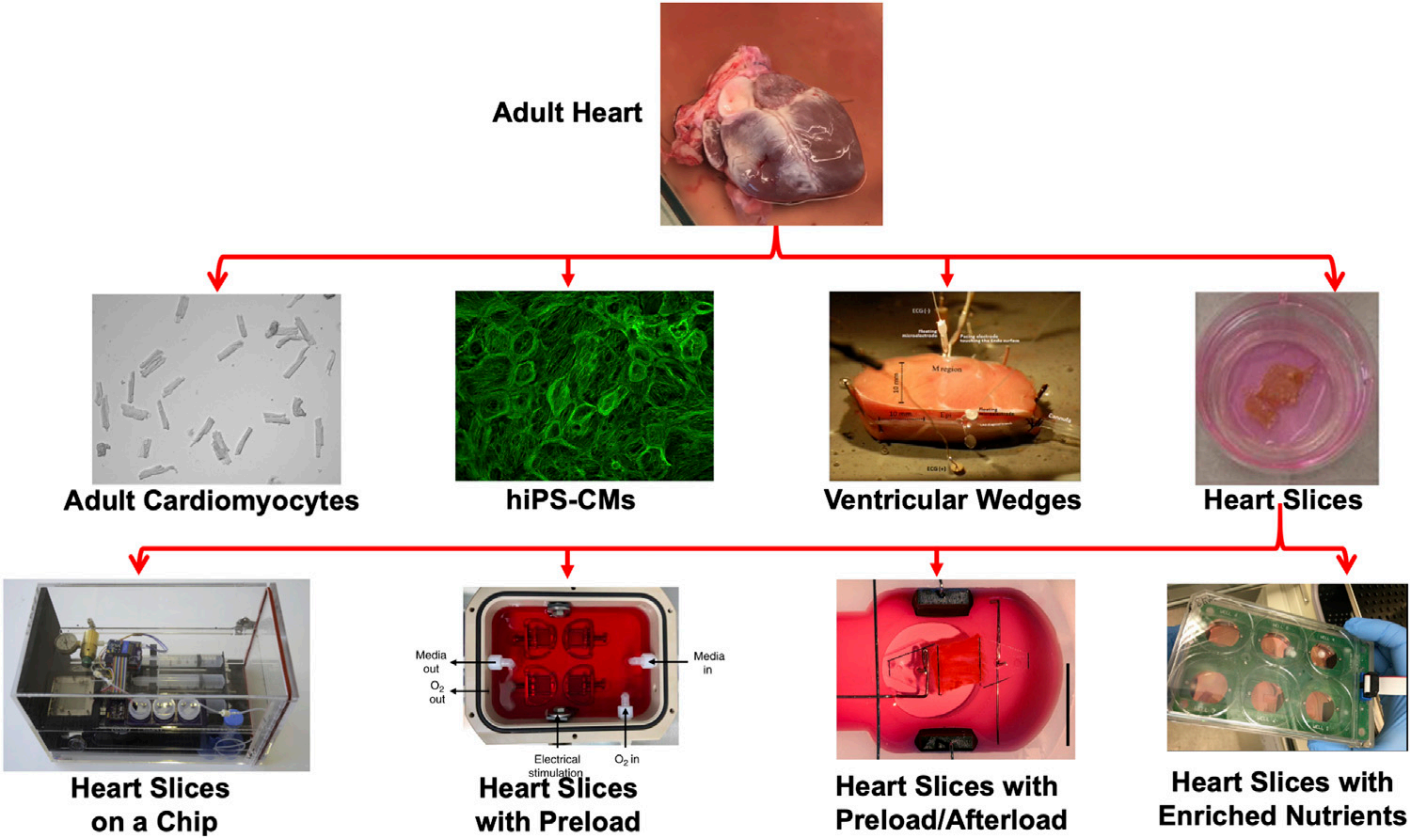
Modeling heart tissue in cultures. Representation of the various platforms used to model cardiac physiology in culture including adult cardiomyocytes (Mohamed et al., 2017), hiPS-CMs (Mohamed et al., 2018), Ventricular wedges (Di Diego et al., 2013) and heart slices (Brandenburger et al., 2012). Recently, several groups advanced the heart slice technology by developing heart slices on-a-chip (Qiao et al., 2019), including preload (Watson et al., 2019), including preload/afterload (Fischer et al., 2019), or enriching the culture medium with nutrients (Ou et al., 2019).

This review article will highlight each of these models and discuss their potential to model cardiac physiology in culture (Figure 1).

## Animal/Human Isolated Adult Cardiomyocyte

One of the oldest and most simplistic cardiac models is the use of isolated human cardiomyocytes. The first isolated cardiomyocyte from adult hearts failed to last for a reasonable time in culture and lacked proper phenotypic characteristics (Harary and Farley, 1963; Halbert and Bruderer, 1971; Halbert et al., 1973). Successful isolation and culture of adult cardiomyocytes were performed first by Powell and Twist in 1976 (Powell and Twist, 1976). Since then, cultured adult isolated cardiomyocytes have been primarily used to study cardiac electrophysiology (Beuckelmann and Wier, 1988; Ke et al., 2007), response to sepsis (Celes et al., 2013), Ca^+2^ dynamics (Beuckelmann et al., 1992; Gorski et al., 2018), gene transfer (Judd et al., 2016), and contractile function (Parikh et al., 1993; Monte et al., 1999). Indeed, the simplistic unicellularity of isolated primary cardiomyocytes provides experimental strength by avoiding extraneous influences from other tissue components (Gilsbach et al., 2014). Moreover, when cultured adhering to a coated plastic surface, they will retain many adult cardiomyocyte phenotype characteristics including cell-cell coupling, t-tubule organization, and rod-shaped morphology (Mitcheson, 1998). While having been instrumental to cellular level research, adult isolated cardiomyocytes’ appropriateness for predicting cardiotoxicity and macrolevel response to drugs and therapies is restricted. Although isolated cardiomyocytes can survive for weeks, the single cell-type culture will rapidly dedifferentiate and lose function within 48 h of culture (Bird et al., 2003). Additionally, the low cell yield with currently used enzymatic isolation protocols and cellular damage limit their use in high throughput screening (Tytgat, 1994; Dipla et al., 1998; Dobrev et al., 2000). Adult isolated primary cardiomyocytes may be unsuitable for long-term drug screening due to their limited life span in culture and its nature of replicating only a single cell type out of the multiplexed heart cell types. Nevertheless, recent efforts have focused on improving isolation protocols to improve cell yield and lowered the damage to the cardiac tissue (Guo et al., 2018). Notably, Callaghan et al. (Callaghan et al., 2020) have shown that Geltrex and blebbistatin using adult mouse cardiomyocytes enhanced survival in culture with maintained cellular function. However, the inhibition of cardiomyocyte contraction using blebbistatin may limit the model’s use to study cardiomyocyte contractility.

## Human-Induced Pluripotent Stem Cell-Derived Cardiomyocytes (hiPSC-CMs)

Human-induced pluripotent stem cells (hiPSC) reprogramming was first demonstrated in 2007 (Takahashi et al., 2007), and hiPSC-cardiomyocytes (hiPSC-CMs) have since been extensively used to determine drug cardiotoxicity, arrhythmogenicity (Sirenko et al., 2013; Pointon et al., 2015; Sharma et al., 2018), and cardiac disease modeling (Jung and Bernstein, 2014; Yang et al., 2015) due to the relative simplicity of hiPSC-CMs generation and culture process (Denning et al., 2016). However, it is well known that hiPSC-CMs are structurally and functionally similar to fetal cardiomyocytes due to their lack of cell-cell coupling, under-developed sarcoplasmic reticulum, and no T-tubules; hence slow excitation-contraction coupling (Denning et al., 2016).

With these apparent limitations in mind, several efforts have been conducted to promote their maturation, discussed in detail in Ahmed et al. review (Ahmed et al., 2020). These studies aim to mimic certain cardiac milieu stimuli using integrated heart-on-chip systems to include extracellular matrix (Roberts et al., 2016), 3-D structure (Zhang et al., 2013; Zhang et al., 2016), electrical (Tandon et al., 2009; Nunes et al., 2013), and electro-mechanical stimulation (Jackman et al., 2016; Ruan et al., 2016; Ronaldson-Bouchard et al., 2018). Notably, Kroll et al., (Kroll et al., 2017) developed a force stretch system that can apply synchronized mechanical stress and electrical stimulation on hiPSC-CMs cultured on a flexible PDMS membrane. They have shown enhanced N-cadherin signal, stress fiber formation, sarcomere shortening, and contractile protein expression with hiPSC-CMs conditioning, indicating a trend toward maturation. We suggest the following comprehensive reviews for details (Benam et al., 2015; Ronaldson-Bouchard and Vunjak-Novakovic, 2018; Zhang et al., 2018; Zhao et al., 2020). These advancements allow the hiPS-CMs to become the most widely used platform for drug screening due to their stability in culture for a long time and their physiological resemblance to the human heart.

## Animal-Derived Arterially Perfused Left Ventricular Wedge Preparations

Arterially perfused left ventricular wedges were developed by Yan and Antzelevitch in 1996 (Yan and Antzelevitch, 1996; Yan et al., 1998) using canine hearts. Since then, ventricular wedge preparations have been expanded to other animal models, including humans, and have been extensively used in studying heart electrophysiology, conduction velocity, and the development of arrhythmia (Chen et al., 2006; Glukhov et al., 2010; Lou et al., 2011; O’Shea et al., 2019). The advantage of this model is producing 3-dimensional tissue-level insight into cardiac physiology and pathophysiology.

However, where ventricular wedges fall short is the relatively low throughput of the preparation due to the coronary system’s complexity and, therefore, low sample yield per heart, (Qiao et al., 2019) limiting their use in drug testing. Furthermore, the preparations can only remain viable for a few hours in perfusion before suffering a similar fate to isolated cardiomyocytes (Kang et al., 2016) restricting their use in subacute and chronic studies. Such a system as a drug screening platform would be challenging due to the short life in culture and the low throughput.

## Animal/Human-Derived Heart Slices to Model Cardiac Physiology

The first thin (<400 μm) heart slices were developed in 1992 (Parrish et al., 1992) in the advent of tissue slicing technology with relatively high precision and reproducible slice thicknesses. In this study, rat ventricular heart slices were cultured for 24 h with maintained viability and metabolism (Parrish et al., 1992). The key feature was the use of continuously oxygenated media while rotating the culture within a cylinder. Brandenburger et al., (Brandenburger et al., 2012) 10 years later developed a more simplistic approach to overcome the inadequacies with isolated cardiomyocytes and ventricular wedge preparations. Brandenburger and colleagues used a transwell membrane to allow for a liquid-air interface.

Thin heart tissue slices (300 μm) have since been shown to achieve high viability, retain typical tissue architecture, contractility, calcium handling, and conduction velocity for 24 h (Kang et al., 2016; Watson et al., 2017). Additionally, heart slices maintain a 3-dimensional structure with the multi cell-type environment and the presence of extracellular matrix (ECM). This allows for other cellular components in the heart slices and ECM to interact with the cardiomyocytes through direct cellular contact or paracrine secretion, which is essential to maintain a mature cardiomyocyte phenotype (Talman and Kivelä, 2018; Yoshida et al., 2018). It is important to note that heart slices and ventricular wedge preparations are different. Thin heart slices could remain viable and functional in culture for 24 h, do not require external perfusion, and obtain oxygen and nutrients directly from culture media. These unique advantages make them adequate for medium to high throughput systems for drug discovery.

Since their inception, heart slices have been prepared from several animals as well as human hearts. Detailed protocols for preparing heart slices have been described by our group and others (Brandenburger et al., 2012; Watson et al., 2017; Watson et al., 2019; Ou et al., 2020). While multiple factors control the maturation and regulation of the adult cardiac phenotype, the structural fidelity of heart slices provides cell-cell interactions, transmembrane regulation and maintains standard tissue stiffness. These heart slices' thinness simplifies nutrient and oxygen diffusion through the tissue and provides a “pseudo” 3-dimensional model that can allow for multiplexed cellular-level studies. Pseudo-3-dimensional model refers to the large surface area to thickness ratio of the heart tissue slices, which does not compromise the tissue's 3-D structure while maintaining proper substrate exchange.

## Recent Advancements in Heart Slices

Early transwell culture of cardiac slices by Brandenburger et al. maintained viability, ß-adrenergic response, and many electrophysiological properties for 28 days (Brandenburger et al., 2012). However, the prolonged culture of heart slices in transwell systems produced an observable loss in the tissue's structural and functional integrity after 24 h in culture shown by a >90% drop in contractility. The observed tissue remodeling reaffirmed this with a dedifferentiated cardiomyocyte phenotype and a significant reduction in mRNA expression of MLC2 and α-actin (Brandenburger et al., 2012). Subsequent efforts used the same culture transwell system to culture heart slices 24 h using human and other vertebrate hearts (Kang et al., 2016; Watson et al., 2017). Indeed, the heterogeneous heart slice preparation incorporates many organotypic characteristics of the myocardium, and they will undergo dedifferentiation due to the non-physiological environment in culture (Parrish et al., 1992; Brandenburger et al., 2012; Perbellini et al., 2018).

The native heart experiences several hemodynamic and neurohormonal factors which precisely compose its ideal environment for functional integrity (Barton et al., 2005). Disruption of this balance in culture will ultimately promote some level of adaptive remodeling within the heart. This highlights the importance of incorporating biomimetic stimulants to maintain cardiac phenotype properly. But given the cardiovascular system’s complexity, it is difficult to define all the cardiac milieu constituents responsible for this balance.

In 2019, several efforts were taken to prolong further the functional viability of the heart slices in culture. The most relevant factors addressed in these efforts are biophysical stimulation through electrical pacing, mechanical loading (preload and afterload), and proper oxygenation and nutrient support (Fischer et al., 2019; Ou et al., 2019; Qiao et al., 2019; Watson et al., 2019).

Electrical impulse propagation in the heart is regulated through the gap junctions, which synchronize action potential progress and contraction (Ye and Black, 2011). The disruption of the gap junction protein connexin 43, localization, or expression can trigger arrhythmias. Continuous electrical stimulation of hiPSCs has been shown to improve connexin abundance and improve sarcomere ultrastructure (Hirt et al., 2014). The other side of the coin is the mechanical loading condition in culture. Mechanical cues in the native heart play an essential role in steering physiological/pathological remodeling such as dilated or hypertrophic cardiomyopathy (Barton et al., 2005). The absence of electrical stimulation, preload, and afterload in static transwell culture may partially explain the loss of contractility and contractile protein expression after 24 h in culture (Brandenburger et al., 2012). Furthermore, the high metabolic demand of the myocardium necessitates the incorporation of rich nutrient media. Several media supplements have been previously used to support hiPSC-CMs in culture (Yamakawa et al., 2015; Mohamed et al., 2017; Mohamed et al., 2018). Likewise, oxygenation of the media has proven crucial to prevent tissue hypoxia since the slice thickness (300–400 μm) approaches the diffusion limit of oxygen of the myocardium and can heavily impact cardiac gene expression (Giordano, 2005).

In this context, we review four recent studies from different laboratories that have made significant efforts to emulate the cardiac milieu (Fischer et al., 2019; Ou et al., 2019; Qiao et al., 2019; Watson et al., 2019).

## Heart Tissue Slice on a Chip Culture Chamber

Qiao et al. (Qiao et al., 2019), developed an automated heart-on-a-chip culture system that uses heart tissue slices and supports accurately controlled culture conditions with continuous O_2_/CO_2_ control, an orbital shaker, media circulation, static mechanical loading, and electrical stimulation. The system was tested using murine atrial and human ventricular slices. The culture system itself offered some unique advantages, including optical mapping, contraction rate using far-field pseudo ECG electrodes, and integrated pacing electrodes. Human heart slices preserved electrophysiology for four days in culture with uniform repolarization, average transverse conduction velocity, and anisotropic conduction. While this culture system is innovative, the study presented did not apply electrical stimulation or mechanical loading in the smart tissue culture chamber. Additionally, it was only used to test the cultured slices’ electrophysiology, which was more stable in early static transwell cultures than the drastic drop in contractility and phenotype on the functional and transcriptional levels in extended culture (Brandenburger et al., 2012). Hence, further validation to confirm any implied reduction in dedifferentiation or maintenance of heart slices functionality in culture is needed.

## Introduction of Preload and Afterload

Watson et al. (Watson et al., 2019), investigated the application of simultaneous electrical stimulation and preload on rat, rabbit, and failing human heart slices. The system used a hand-operated screw and nut post to progressively stretch the tissue to different sarcomeric lengths and achieved sub-micrometer resolution. The study compared this system to static transwell culture and fresh tissue. Rat heart slices cultured for 24 h with an increased sarcomeric length of 2.2 μm showed maintained contractile function, normal electrophysiology, conduction velocity, calcium handling, and action potential compared to fresh tissue. Interestingly, this model showed an upregulation of hypertrophic genes and increased calcium transient amplitude within 24 h in culture compared to fresh heart slices. At the same time, cardiomyocyte size did not vary for all preloading conditions tested. Five days culture with rabbit heart showed no deterioration in contractile function. Heart failure slices from human hearts showed some degree of preserved contractility after 24 h in culture.

There are obvious limitations when using slices from rat hearts since their high metabolic rates make them more susceptible to dedifferentiation. Moreover, the short culture time leaves unanswered questions regarding the long-term performance of static preload. Nevertheless, the improvement in functionality with stretching heart slices underlined the undeniable mechanical stimulation role in regulating the cardiac phenotype *in vitro*. Additionally, the upregulation seen in some hypertrophic genes may be related to the isometric contraction of the tissue slices with theoretically infinite afterload (Pitoulis et al., 2020).

A reasonably similar system was developed by Fischer et al. (Fischer et al., 2019). In this system, slices from normal and failing human hearts were stretched between two posts in culture with electrical stimulation. The heart slice was fixed at one end, while the other end was connected to a spring cantilever to provide a linear mechanical afterload. A small magnet coupled with a magnetic field sensor was used to detect the movement of the free end of the tissue slice, and the contractile force was calculated in real-time using the spring constant. While using a low stimulation pace (0.2 Hz) for up to 4 months, the study showed preserved contractility, preserved tissue elasticity, connexin localization, and α-actinin. But there is severe downregulation of cardiac gene expression at the first assessed time point (8 days). Unlike the culture system by Watson et al. (Watson et al., 2019), this system allowed for active tissue contraction and shortening. Nevertheless, as noted by Pitoulis et al. (Pitoulis et al., 2020) this mode of contraction is still short of the physiological force-length loop where relaxation and contraction are separated by periods of isometric relaxation and contraction.

## Optimization of Nutrients and Electrical Stimulation

Our group has shown that through optimized proprietary culture media, frequent oxygenation, and electrical stimulation, tissue slices can preserve viability, functionality, and cardiomyocyte structure for up to 6 days (Ou et al., 2019; Ou et al., 2020). The 300 µm human and pig heart slices were cultured at a physiological rate (1.2 Hz, 72 beats/min) and showed normal viability, gene expression profile, calcium homeostasis, contractility, twitch force generation, and response to ß-adrenergic stimulation over the first six days. However, by day 10, dedifferentiation was evident by the disruption of gene expression of more than 500 genes, connexin-43 delocalization, and loss of contractility. Nonetheless, prolonging functionality and maintaining cardiac phenotype for six days is a significant advancement compared to the early transwell culture systems. However, the critical element missing is the addition of synchronized mechanical loading of the heart tissue.

## Applications of Heart Slices as a Cardiotoxicity Screening Platform

To date, the perfect human cardiac tissue model does not exist. The suitability of the testing platform is dependent on the level of complexity and experimental question which needs to be answered for each specific application. Engineered 3-dimensional tissue culture of hiPSC-CMs have shown promise in producing heterogeneous, relatively mature cardiac constructs that suit a wide range of applications (e.g., regenerative medicine, personalized assays, and basic science) (Lemoine et al., 2017; Goldfracht et al., 2019; Ahmed et al., 2020). Nevertheless, the recent advancements in biomimetic heart slice technology discussed above show that this technology is progressing in the right direction and may soon provide a medium/high throughput platform to recapitulate drug effects on myocardial mechanics, electrophysiology at a cellular level that does not compromise organ level complexity.

The most immediate application of organotypic heart slices is in the field of drug screening and cardiotoxicity. Current preclinical cardiotoxicity systems are failing to detect the cardiotoxicity of many drugs (Gwathmey et al., 2009). We have recently shown that our optimized heart slice system (Ou et al., 2019) can reliably detect drug-related cardiotoxicity (Miller et al., 2020). Three medications from three different anticancer categories were tested (doxorubicin, trastuzumab, and sunitinib). Cardiac slices demonstrated toxic effects in response to all treatments and were superior to hiPSC-CMs in detecting sunitinib at 0.1–1 μM concentration. These results provide evidence of the clinical relevance of heart slices as an essential tool in drug development.

## Current Limitations and Future Perspective of the Cardiac Slice Technology

There are several limitations for the heart slice culture, which will need to be addressed in further studies. First, the exact nutrients needed to further maintain the metabolic activities and mitochondrial utilization rates in the presence of several nutrients such as fatty acids, glucose, fructose, corticosteroids, and hormones of heart slices. Our lab has performed the only study to provide a proof of concept study to show that heart slices can reflect clinical phenotypes of cardiotoxins (Miller et al., 2020). This study used three known cardiotoxins at three different concentrations and assessed effects at one time point (48 h). Further studies are needed to address a more in-depth time course and test other cardiotoxins and cardiac therapeutics. Furthermore, studies to test cardiotoxicity in enhanced cardiac function models are required.

Another limitation of the heart slices is the inability to record conduction velocity and electrophysiological properties within the culture. Considering that electrophysiology is highly relevant for predicting arrhythmogenic drug potential, action potential recordings from the whole tissue would be informative. In comparison, Integrated and miniaturized technologies that use hiPSC-CMs, such as heart-on-a-chip systems, have been used to record accurate electrophysiological data and other quantitative data due to the ease with which microsensors can be integrated within the system (Cohen-Karni et al., 2009; Nunes et al., 2013; Stoehr et al., 2016). Indeed, a recent study established a glass microelectrode technique to perform patch clamping on the whole heart (Barbic et al., 2017). However, further research is required to adapt this microelectrode technology for recording action potentials in heart slices. Another limitation is that heart slice technology is technically and financially demanding, limiting its general or widespread use. To this end, several groups have recently published detailed video protocols to promote this technology (George et al., 2020; Ou et al., 2020). Another major limitation of the heart slice technology is the relatively short functional viability in culture (6 days), making it suitable for only acute and subacute cardiotoxicity testing but not for chronic toxicity testing. Further efforts to prolong the viability in culture are needed. Moreover, access to fresh human heart tissue is limited to a few research groups, limiting the spread of the technology. Research for preserving the human heart tissue should be conducted to facilitate the spread of the technology.
